# Supplementary material for: Access to radiotherapy in improving gastric cancer care quality and equality
Source: Commun Med (Lond). 2024 Nov 2;4:225. doi: 10.1038/s43856-024-00655-z (PMC11531536; doi:10.1038/s43856-024-00655-z)
Supplement: Supplementary file 2 — Supplementary Information [file 43856_2024_655_MOESM2_ESM.pdf]

Access to Radiotherapy in Improving Gastric Cancer Care Quality and Equality

Minmin Wang, Kepei Huang, Xiaohan Fan, Jia Wang, Yinzi Jin, Zhi-Jie Zheng

Supplemental Material

Supplemental Table 1. Definition and coding forms of variables used in association analysis.

Supplemental Table 2. Global burden of gastric cancer in 1990 and 2019, by sexes.

Supplemental Figure 1. Age trend of GDR of gastric cancer QCI by SDI regions.

Supplemental Table 1. Definition and coding forms of variables used in association analysis.

Variable	Definition	Data source	Coding form
Gender-specific QCI	As defined in the manuscript	GBD dataset	Log-transformed
GDR of gastric cancer QCI	Ratio of the QCI score in women divided by that in men	GBD dataset	Absolute value of log-transformed
Radiotherapy unit density	Number of radiotherapy units, including the following: Linear Accelerators, Cobalt-60, Brachitheraphy, x-ray generator, and ion proton radiotheraphy equipment from the public and private sectors, per 1 000 000 population.	GHO dataset	Log-transformed
Number of medical doctors	Medical doctors per 10000	GHO dataset	Log-transformed
Hospital beds density	Hospital beds (per 10 000 population)	GHO dataset	Log-transformed
Sociodemographic index	The SDI is a geometric average of 0 to 1 in each country or region. It is obtained by combining the total fertility rate of women younger than 25 years, the education level of people aged 15 years and older, and the lag in the per capita income distribution.	GBD dataset	Five levels by SDI: high (> 0.81), high-middle (0.70–0.81), middle (0.61–0.69), low-middle (0.46–0.60), and low (< 0.46).
Universal health services	Coverage of essential health services (defined as the average coverage of essential services based on tracer interventions that include reproductive, maternal, newborn and child health, infectious diseases, non-communicable diseases and service capacity and access, among the general and the most disadvantaged population). The indicator is an index reported on a unitless scale of 0 to 100, which is computed as the geometric mean of 14 tracer indicators of health service coverage.	GHO dataset	Continuous variable

Supplemental Table 2. Global burden of gastric cancer in 1990 and 2019, by sexes.

**Measure**	**Gastric cancer burden in 1990 (95% UI)**	**Gastric cancer burden in 2019 (95% UI)**	**Annual rate of change (%) (95% UI)**
**Both**			
Deaths	20.48 (19.25,21.62)	11.88 (10.82,12.82)	-0.42 (-0.47, -0.36)
DALYs	493.38 (463.73,523.70)	268.40 (245.49,290.61)	-0.46 (-0.51, -0.40)
YLDs	5.52 (4.02,7.04)	4.25 (3.08,5.57)	-0.23 (-0.31, -0.14)
YLLs	487.86 (458.96,517.98)	264.15 (241.47,286.03)	-0.46 (-0.51, -0.40)
Prevalence	36.05 (34.31,37.68)	32.83 (29.48,36.53)	-0.09 (-0.18, 0.02)
Incidence	22.44 (21.21,23.59)	15.59 (14.11,17.15)	-0.31 (-0.37, -0.23)
**Male**			
Deaths	27.62 (25.53,29.78)	16.59 (14.8,18.34)	-0.40 (-0.47, -0.32)
DALYs	654.98 (602.33,706.87)	368.85 (328.19,410.33)	-0.44 (-0.51, -0.35)
YLDs	7.50 (5.48,9.66)	6.17 (4.43,8.11)	-0.18 (-0.29, -0.04)
YLLs	647.48 (594.89,699.54)	362.68 (322.15,403.44)	-0.44 (-0.51, -0.36)
Prevalence	49.18 (46.26,52.04)	48.63 (42.40,55.87)	-0.01 (-0.14, 0.15)
Incidence	30.42 (28.41,32.49)	22.39 (19.8,25.34)	-0.26 (-0.35, -0.15)
**Female**			
Deaths	14.68 (13.59,15.72)	7.92 (7.07,8.76)	-0.46 (-0.51, -0.39)
DALYs	350.87 (324.01,377.45)	178.25 (160.52,196.94)	-0.49 (-0.54, -0.42)
YLDs	3.84 (2.79,4.87)	2.55 (1.83,3.35)	-0.33 (-0.40, -0.25)
YLLs	347.03 (320.05,373.69)	175.7 (157.9,194.11)	-0.49 (-0.55, -0.42)
Prevalence	24.62 (23.12,25.99)	18.66 (16.57,20.84)	-0.24 (-0.32, -0.15)
Incidence	15.81 (14.72,16.82)	9.71 (8.67,10.72)	-0.39 (-0.45, -0.31)

DALY, disability adjusted life years; YLD, years lived with disability; YLL, years of life lost; UI, uncertainty interval.

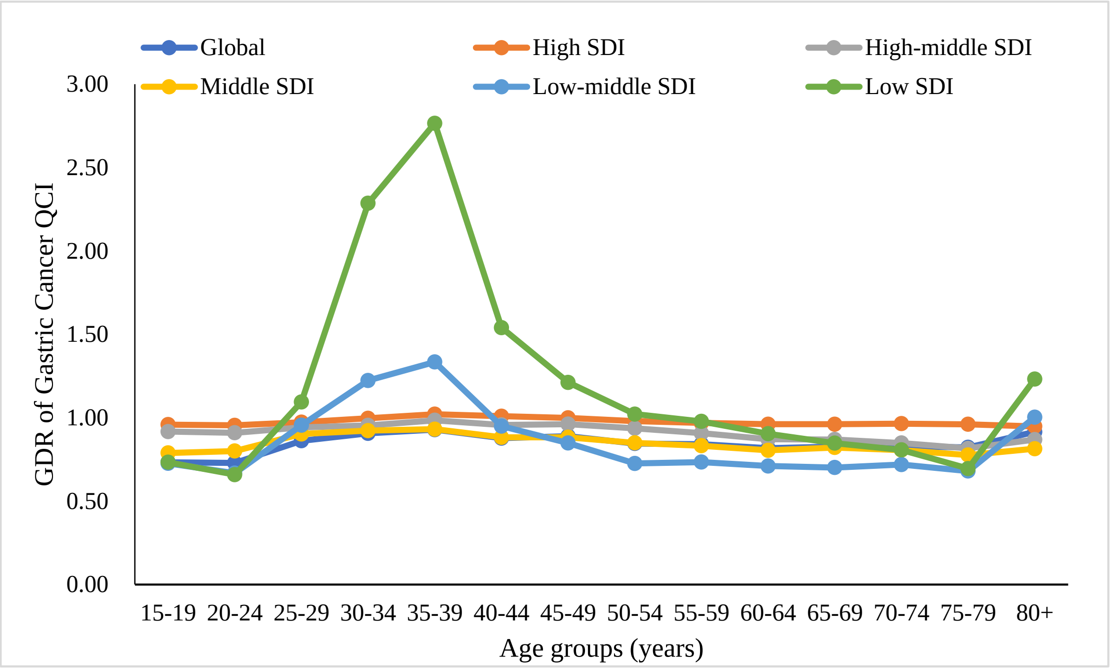

Supplemental Figure 1. Age trend of GDR of gastric cancer QCI by SDI regions. GDR, gender difference ratio; QCI, quality of cancer care; SDI, sociodemographic index.
