# Supplementary material for: Access to radiotherapy in improving gastric cancer care quality and equality
Source: Commun Med (Lond). 2024 Nov 2;4:225. doi: 10.1038/s43856-024-00655-z (PMC11531536; doi:10.1038/s43856-024-00655-z)
Supplement: Supplementary file 3 — Description of Additional Supplementary Files [file 43856_2024_655_MOESM3_ESM.pdf]

Description of Additional Supplementary Files

File name: Supplementary Data 1

File description: The source data is located in Supplementary Data 1.
