# Supplementary material for: Access to radiotherapy in improving gastric cancer care quality and equality
Source: Commun Med (Lond). 2024 Nov 2;4:225. doi: 10.1038/s43856-024-00655-z (PMC11531536; doi:10.1038/s43856-024-00655-z)
Supplement: Supplementary file 5 — Reporting Summary [file 43856_2024_655_MOESM5_ESM.pdf]

nature portfolio

Corresponding author(s): Yinzi Jin

Last updated by author(s): Apr 29, 2024

Reporting Summary

Nature Portfolio wishes to improve the reproducibility of the work that we publish. This form provides structure for consistency and transparency in reporting. For further information on Nature Portfolio policies, see our Editorial Policies and the Editorial Policy Checklist.

Statistics

For all statistical analyses, confirm that the following items are present in the figure legend, table legend, main text, or Methods section.

n/a	Confirmed	
☐	☒	The exact sample size (*n*) for each experimental group/condition, given as a discrete number and unit of measurement
☒	☐	A statement on whether measurements were taken from distinct samples or whether the same sample was measured repeatedly
☐	☒	The statistical test(s) used AND whether they are one- or two-sided<br>*Only common tests should be described solely by name; describe more complex techniques in the Methods section.*
☐	☒	A description of all covariates tested
☐	☒	A description of any assumptions or corrections, such as tests of normality and adjustment for multiple comparisons
☐	☒	A full description of the statistical parameters including central tendency (e.g. means) or other basic estimates (e.g. regression coefficient) AND variation (e.g. standard deviation) or associated estimates of uncertainty (e.g. confidence intervals)
☐	☒	For null hypothesis testing, the test statistic (e.g. *F*, *t*, *r*) with confidence intervals, effect sizes, degrees of freedom and *P* value noted<br>*Give P values as exact values whenever suitable.*
☒	☐	For Bayesian analysis, information on the choice of priors and Markov chain Monte Carlo settings
☒	☐	For hierarchical and complex designs, identification of the appropriate level for tests and full reporting of outcomes
☒	☐	Estimates of effect sizes (e.g. Cohen's *d*, Pearson's *r*), indicating how they were calculated

Our web collection on statistics for biologists contains articles on many of the points above.

Software and code

Policy information about availability of computer code

Data collection	The datasets generated and/or analyzed during the current study are available in the Global Health Data Exchange (http://ghdx.healthdata.org) and Global Health Observatory (https://www.who.int/data/gho). No software was used.
Data analysis	All statistical analyses for this study were created using R v4.1.3 software (http://www.r-project.org/). All tests were two-sided, and P values < 0.05 were considered statistically significant.

For manuscripts utilizing custom algorithms or software that are central to the research but not yet described in published literature, software must be made available to editors and reviewers. We strongly encourage code deposition in a community repository (e.g. GitHub). See the Nature Portfolio guidelines for submitting code & software for further information.

Data

Policy information about availability of data

All manuscripts must include a data availability statement. This statement should provide the following information, where applicable:

- Accession codes, unique identifiers, or web links for publicly available datasets
- A description of any restrictions on data availability
- For clinical datasets or third party data, please ensure that the statement adheres to our policy

The datasets generated and/or analyzed during the current study are available in the Global Health Data Exchange (http://ghdx.healthdata.org) and Global Health Observatory (https://www.who.int/data/gho).

Human research participants

Policy information about studies involving human research participants and Sex and Gender in Research.

Reporting on sex and gender	We reported the gender-specific estimates and the gender disparity of the quality of gastric cancer care. This estimation was retracted from GBD study 2019.
Population characteristics	In this study we described the trend of quality of gastric cancer care by geographic regions and social development levels.
Recruitment	This study did not include human participants.
Ethics oversight	This study did not include human participants and ethic approval was not applicable.

Note that full information on the approval of the study protocol must also be provided in the manuscript.

Field-specific reporting

Please select the one below that is the best fit for your research. If you are not sure, read the appropriate sections before making your selection.

☐ Life sciences ☒ Behavioural & social sciences ☐ Ecological, evolutionary & environmental sciences

For a reference copy of the document with all sections, see nature.com/documents/nr-reporting-summary-flat.pdf

Behavioural & social sciences study design

All studies must disclose on these points even when the disclosure is negative.

Study description	Quantitative design
Research sample	This study was based on the GBD 2019 study, which assessed the disease burden of 369 different diseases and 87 risk factors in 204 countries, 21 regions, and seven super-regions, aiming to provide a comprehensive and comparable global health assessment
Sampling strategy	We included 204 countries and territories in the world.
Data collection	The Global Burden of Disease (GBD) study assessed the disease burden of 369 different diseases and 87 risk factors in 204 countries, 21 regions, and seven super-regions, aiming to provide a comprehensive and comparable global health assessment. In this study, we searched the GBD 2019 database and collected data on gastric cancer incidence, prevalence, mortality, years of life lost (YLLs), years lived with disability (YLDs), and disability-adjusted life-years (DALYs) from 1990 to 2019.
Timing	In this study, we searched the GBD 2019 database and collected data on gastric cancer incidence, prevalence, mortality, years of life lost (YLLs), years lived with disability (YLDs), and disability-adjusted life-years (DALYs) from 1990 to 2019.
Data exclusions	No data was excluded from this current study.
Non-participation	No participants were dropped out.
Randomization	Participants were not allocated to an experimental group.

Reporting for specific materials, systems and methods

We require information from authors about some types of materials, experimental systems and methods used in many studies. Here, indicate whether each material, system or method listed is relevant to your study. If you are not sure if a list item applies to your research, read the appropriate section before selecting a response.

Materials & experimental systems

n/a	Involved in the study
☒	☐ Antibodies
☒	☐ Eukaryotic cell lines
☒	☐ Palaeontology and archaeology
☒	☐ Animals and other organisms
☒	☐ Clinical data
☒	☐ Dual use research of concern

Methods

n/a	Involved in the study
☒	☐ ChIP-seq
☒	☐ Flow cytometry
☒	☐ MRI-based neuroimaging
